# An Account of Uncommon Symptoms Succeeding the Measles; with Some Additional Remarks on the Infection of Measles and Small Pox

**Published:** 1790

**Authors:** James Lucas

**Affiliations:** one of the Surgeons of the General Infirmary at Leeds


### THE LONDON MEDICAL JOURNAL.

I. *An Account of uncommon Symptoms ſucceeding the Meaſles; with ſome additional Remarks on the Infection of Meaſles and Small Pox. By Mr.* James Lucas, *one of the Surgeons of the General Infirmary at Leeds.*

AN unmarried woman, aged twenty-three years, was ſeized with a fever July 21ſt, 1790. On the 23d, in the evening, the meaſles began to appear, and on the 28th the eruption ſeemed to be gradually declining. On the 29th, in the evening, ſhe perceived that ſhe was deprived of the uſe of her lower limbs, although ſhe had not any other uncommon complaint. During the eruption ſhe had menſtruated, but the diſcharge and the time were not in any reſpect unuſual. The following morning ſhe could not paſs any urine, and an obſtinate conſtipation ſucceeded, which was ſcarcely to be overcome by the moſt powerful cathartics, adminiſtered not only by the mouth, but alſo by clyſters. Caſtor oil ſeemed to claim a preference to any other purgative that was employed. The tobacco clyſter ſeems, in ſuch caſes of of debility, to be ſomewhat objectionable, although in inflammatory affections I have found it efficacious, not only as an aperient, but alſo as an antiphlogiſtic remedy.

She had little pain: her chief complaint was a ſenſe of fulneſs in the abdomen. The warm bath, which was directed as a ſtimulus to the viſcera, was followed by a ſlight pain in the legs. Although the uneaſineſs was of ſhort duration, yet it ſeemed to prognoſticate farther advantage from perſiſting in the uſe of the bath: it was, therefore, directed to be employed of an agreeable heat, and for the ſpace of ten minutes, every other night. The pain appeared to be increaſed after every bathing, which proved of eſſential ſervice in producing a feeling in the limbs.

During ten days no urine was evacuated, except what eſcaped upon moving her, or was drawn off by the catheter, which was daily employed; for although the attendants ſeemed to be of opinion that a ſufficient quantity occaſionally and accidentally flowed, yet the intumeſcence in the region of the bladder clearly pointed out the neceſſity of more effectually removing its diſtention. She neither paſſed urine at the time of fœcal diſcharges, nor had any voluntary inclination to void it until her limbs had

ſo

ſo far recovered that ſhe was able to move over the floor.

In ſuch caſes too ſupine a poſture may be pernicious; and endeavours ſhould be uſed, by the patient, to void urine about the time that it has been accuſtomed to be extracted by the catheter.

In every inſtance, but more eſpecially when the feelings of the patient are imperfect, it is neceſſary to diſtinguiſh between an involuntary flowing of urine, and an evacuation under the direction of the patient; for if the bladder is never ſufficiently emptied, its tone will be much longer in being recovered, although the ſwelling in the abdomen ſhould not be of a very painful kind. It would be a fortunate circumſtance if women, in ſuch unhappy caſes, could accompliſh the introduction of the catheter themſelves.

The patient was a week before ſhe could move a limb, and yet in two days more ſhe was able to walk over the floor without much aid. Occaſional aperients and a preparation of bark, together with the warm bath, were the only means employed. Every potent remedy was purpoſely delayed, until it was ſeen what nature's aſſiſtance would accompliſh, as the paralyſis ſeemed to ariſe from debility.

During

During her indifpofition fhe was confined to a large chamber with a north afpect, and in a fine fituation in the country; fhe was neither blooded nor bliftered, nor were any powerful medicines given, to which fuch fingular affection could be afcribed.

Could menftruation at that period have occafioned fuch temporary palfy? — I have known the fame evacuation, during eruptive fevers, occur without any bad confequence. An adult and a child paffed through the difeafe in the fame family, and about the fame time, without any remarkable fymptoms. The patient herfelf obferved, that after the fmall pox, about nine years ago, fhe had been fomewhat fimilarly affected, and had gradually recovered. For a week or two fhe complained of pain in her back, efpecially upon motion.

Several months ago, the hooping cough, fince that time the fmall pox, and now the meafles, have become very prevalent in this town and neighbourhood. The meafles have, in general, been of a favourable kind. I have not feen one inftance of a fatal termination. Great debility has, in many cafes, fucceeded. I bled one patient, and found his blood of a loofe texture; but his recovery appeared to me fomewhat fomewhat impeded, although at the time his fymptoms feemed to require fuch an evacuation.

The eruption has commonly been copious, and in many accompanied with great fever.

Perfons in a country fituation have paffed through the diforder in a more favourable manner than thofe in the town.

An opinion has been maintained, that the fmall pox is not infectious naturally previous to maturation; but an inftance has appeared of the meafles being infectious during the eruptive fever. A woman, who had no fufpicion of any fuch infection about her, took her child with her to pay a vifit to a family in the country. This child having been obferved by the family to have a cough, and to be ill, its mother was requefted to take it away, and reminded that the fmall pox and meafles were at Leeds, from whence fhe came. The child was accordingly removed, and the next morning had an eruption, which proved to be the meafles. On the fourteenth day from the removal of this patient three children of the family, who refided in the country, and alfo a man fervant, all of whom had been clofe to the child that had been

 taken taken there upon a viſit, ſickened, and had the meaſles. Three other children, two of whom were the youngeſt, and had not been equally near the child, received the infection from their ſiſters. They all had the diſorder in as moderate a degree as any that I have attended.

A young lady was ſenſible of a bad ſmell, and complained of being ſick, ſoon after ſhe diſcovered that ſhe had been near a child with the ſmall pox. In about three weeks the eruption in herſelf was as copious from head to foot as I ever ſaw, and ſhe died on the fourth day, before there were any ſigns of maturation. Her eldeſt ſiſter, who had been cautiouſly prevented from ſeeing the patient during her life, imprudently ſaluted the corpſe, and with great hazard paſſed through the diſorder. I have no doubt but that inoculation might, in each of theſe caſes, have ſuperſeded the natural infection, had it been done even ſome days after the natural infection had been received; and I am inclined to think that in ſo dangerous a caſe, as the former, cold bathing might be warrantable, as petechiæ were very numerous. In the caſe of a weakly child, who had a copious eruption of ſmall pox, I once ſaw the cold bath uſed, accidentally, without any apparent harm.

Although

Although under natural infection the fmall pox and meafles may be, during the eruptive fever, infectious, yet it is moft probable that when thefe difeafes are milder, or artificially received, there would at fuch times be lefs danger of contagion. The cuftom of perfons unneceffarily vifiting infectious corpfes, and inviting numbers to fuch funerals, are certainly of more ferious confequence than is generally imagined.

*Leeds,*
Auguft 22, 1790.

